# Evaluating the Efficacy of a Thermoresponsive Hydrogel for Delivering Anti-Collagen Antibodies to Reduce Posttraumatic Scarring in Orthopedic Tissues

**DOI:** 10.3390/gels9120971

**Published:** 2023-12-12

**Authors:** Andrzej Steplewski, Jolanta Fertala, Lan Cheng, Mark L. Wang, Michael Rivlin, Pedro Beredjiklian, Andrzej Fertala

**Affiliations:** 1 Department of Orthopaedic Surgery, Sidney Kimmel Medical College, Thomas Jefferson University, Philadelphia, PA 19107, USA; 2 Department of Neurosciences, Sidney Kimmel Medical College, Thomas Jefferson University, Philadelphia, PA 19107, USA; 3 Rothman Institute of Orthopaedics, Thomas Jefferson University Hospital, Philadelphia, PA 19107, USA

**Keywords:** therapeutic antibody, fibrotic scar, orthopedic tissues, thermoresponsive hydrogel, peripheral nerve, arthrofibrosis, collagen fibrils

## Abstract

Excessive posttraumatic scarring in orthopedic tissues, such as joint capsules, ligaments, tendons, muscles, and peripheral nerves, presents a significant medical problem, resulting in pain, restricted joint mobility, and impaired musculoskeletal function. Current treatments for excessive scarring are often ineffective and require the surgical removal of fibrotic tissue, which can aggravate the problem. The primary component of orthopedic scars is collagen I-rich fibrils. Our research team has developed a monoclonal anti-collagen antibody (ACA) that alleviates posttraumatic scarring by inhibiting collagen fibril formation. We previously established the safety and efficacy of ACA in a rabbit-based arthrofibrosis model. In this study, we evaluate the utility of a well-characterized thermoresponsive hydrogel (THG) as a delivery vehicle for ACA to injury sites. Crucial components of the hydrogel included N-isopropylacrylamide, poly(ethylene glycol) diacrylate, and hyaluronic acid. Our investigation focused on in vitro ACA release kinetics, stability, and activity. Additionally, we examined the antigen-binding characteristics of ACA post-release from the THG in an in vivo context. Our preliminary findings suggest that the THG construct exhibits promise as a delivery platform for antibody-based therapeutics to reduce excessive scarring in orthopedic tissues.

## 1. Introduction

Posttraumatic scarring of orthopedic tissues is a severe medical and socioeconomic problem. The most comprehensive data on the prevalence of this disorder are related to total knee arthroplasty (TKA), a procedure for alleviating pain and restoring function in degenerative knee joint diseases. However, a complication of TKA is arthrofibrosis, or joint stiffness, which affects several joints, including the knee, shoulder, elbow, and hand. Arthrofibrosis necessitates aggressive physical therapy or revision surgery to restore joint motion [1]. These procedures increase the cost of TKA and the risk of infection, inflammation, and post-revision fibrosis [1,2]. More than 700,000 TKA surgeries are performed annually in the U.S. Estimates suggest that 3–12% of TKA patients develop arthrofibrosis [2]. Arthrofibrosis is responsible for 28% of hospital readmissions due to surgical complications within 90 days of discharge and 10% of all revisions within 5 years of the initial surgery [2]. About 25% of patients treated with motion-restoring surgical procedures still require multiple surgeries, with only 37% reporting satisfactory results [2]. Studies indicate that if the number of TKA surgeries continues to increase at the current rate, by 2030, the demand for this procedure will exceed 4 million per year in the U.S.. Similarly, TKA revisions are projected to increase by over 600% compared to 2003, resulting in a potential total cost of about $35 billion per year for revised TKA by 2030 in the U.S. alone [3].

In some patients, manipulation under anesthesia (MUA), a procedure in which a surgeon performs a series of forceful flexions and extensions of the stiff joint that may break fibrotic tissue and improve mobility. However, MUA is not fully effective and requires steroids to reduce inflammation and avoid additional fibrotic responses [4]. Moreover, MUA significantly increases the risk of bone and cartilage fractures, ectopic ossification, and hematoma [1]. Remarkably, MUA was described in 1872 by Duplay as a treatment for stiff shoulders [5]. It persists today not due to its efficacy but because there are no adequate and safe alternatives to reduce arthrofibrosis.

Orthopedic injuries are often associated with peripheral nerve damage, resulting in scarring that causes pain and severely limits vital functions of the innervated sites. About 7% to 20% of patients who undergo primary median nerve release report pain and symptom recurrence [6]. Scar-related nerve compression symptoms persist after 40% to 90% of revision procedures, and 20% of patients require a third operation [7]. Approximately 25% of surgeries to release the ulnar nerve fail outright, and the original symptoms reoccur in 75% of patients who undergo this surgery [8]. Overall, traumatic damage to peripheral nerves with devastating consequences represents 2% to 3.3% of all extremity trauma [9,10]. Up to 33% of all peripheral nerve injuries exhibit incomplete nerve recovery and poor functional outcomes, including the loss or only partial recovery of motor and sensory functions, chronic pain, muscle atrophy, and profound weakness [9]. Consequently, these problems can lead to the loss of normal limb function, lifelong morbidity, and permanent disability. In addition to nerve damage resulting from accidental injuries (87%), surgeries and tissue removal (e.g., tumors) can also damage the peripheral nerves (12%) [11]. Nerve injuries are often associated with high socioeconomic costs due to prolonged rehabilitation and absenteeism of young trauma victims. For instance, hospital charges associated with treating peripheral nerve injuries of the upper extremities increased from 2001 to 2013, representing a compound annual growth of 9.6% [12].

Despite differences in the architecture and composition of tissues associated with arthrofibrosis and neural scarring, these disorders are all caused by excessive production of collagen-rich fibrotic tissue. Research has demonstrated that inflammation drives this production, promoting the synthesis of profibrotic growth factors and stimulating the proliferation of profibrotic cells [13]. Most therapeutic approaches to reducing posttraumatic scarring in orthopedic tissues have focused on targeting profibrotic cells, broad inflammatory processes, and their regulators. For instance, inflammatory cells that drive fibrotic healing include mast cells and macrophages [14]. However, recent clinical trials have shown that ketotifen, a mast cell stabilizer, does not significantly reduce posttraumatic elbow stiffness [15]. Similarly, blocking profibrotic transforming growth factor β1 (TGFβ1) was evaluated as an anti-scarring treatment for injured nerves [16]. Due to safety concerns, pursuing this target did not lead to the development of therapies that could reduce the scarring of peripheral nerves. Scientists have also explored blocking calcium channels to reduce collagen production in a rat sciatic nerve injury model [17]. Nevertheless, the utility of this approach has not been thoroughly scrutinized in human patients thus far, so its clinical value is unknown.

Studies have shown that despite their anatomical location, collagen I-based fibrils constitute the bulk of scar mass in many healing tissues. For example, collagen I makes up over 90% of the scar mass formed in the posterior capsules after a knee injury in the arthrofibrosis rabbit model [18]. Similarly, our studies of fibrotic tissue formed after sciatic nerve injury indicated robust formation of collagen fibril-rich deposits [19]. Consequently, our group has developed an antifibrotic approach targeting collagen fibril formation [20]. We have rationally engineered a humanized monoclonal antibody (ACA) that targets a specific domain of collagen I, namely the C terminal telopeptide of the α2(I) chain (α2(I)Ct) [20]. Subsequently, we demonstrated that ACA binding to this target prevents the aggregation of collagen molecules into fibrils. Furthermore, we have established the safety and anti-scarring utility of ACA in a rabbit-based arthrofibrosis model [21]. In these early studies, we applied subcutaneous programmable pumps to deliver ACA to injury sites [21]. While this method has effectively delivered ACA, it is not readily applicable in the clinical environment.

Consequently, we hypothesized that an appropriate hydrogel (HG) could be an effective ACA delivery platform. To explore the delivery of ACA to treat excessive scar formation in orthopedic tissues, we investigated the compatibility of ACA with a well-characterized thermoresponsive hydrogel (THG) in vivo.

## 2. Results and Discussion

Our results showed that the THG construct offers suitable ACA release kinetics, maintains ACA integrity, and does not interfere with its antigen-binding activity. Using an in vivo nerve injury model, we also demonstrated that the construct does not cause side effects at its injection sites. Overall, our exploratory research using a rabbit nerve injury model indicates that the THG material offers an effective platform for delivering ACA to treat excessive scar formation in orthopedic tissues. Future studies will refine the timing of ACA application and its dosage regimens to achieve optimal antifibrotic results.

The motivation for testing the utility of THG in delivering ACA to injury sites stemmed from the necessity of having a relevant method for the local application of this therapeutic antibody. The local application of therapeutic antibodies offers several advantages over systemic delivery. Firstly, it enables the attainment of higher antibody concentrations at the injury sites. Secondly, it minimizes the potential risks of side effects, such as off-target binding. Thirdly, it creates local depots capable of releasing therapeutic antibodies over extended periods, thereby reducing the need for frequent antibody injections. These advantages of local antibody delivery can potentially enhance efficacy and lower treatment costs [22].

We defined the essential criteria for an ACA delivery material, encompassing appropriate antibody release profiles, injectability, biodegradability, and nontoxicity. In our case, the material’s mechanical properties were not a primary concern because the excessive scar formation we aimed to target in orthopedically relevant tissues occurs in their soft elements.

After an extensive literature review, we identified a thermoresponsive hydrogel material initially designed for delivering therapeutic antibodies to the eye [23]. A similar material was also tested for safe antibody delivery to orthopedic tissues, thus presenting a potentially promising ACA delivery platform [24,25]. The safety of similar thermoresponsive hydrogels as a vehicle to deliver drugs, including antibodies, has already been established [26,27,28]. As a result, we opted to utilize this thoroughly characterized thermoresponsive hydrogel in this study, aiming to assess its compatibility with ACA.

### 2.1. ACA Production

Following our established protocols, we utilized a humanized immunoglobulin G4 (IgG4) version of ACA produced by Chinese hamster ovary (CHO) cells [29]. The ACA batch employed in this study met the critical criteria for purity, integrity, and binding specificity [29].

### 2.2. ACA–THG Characteristics

#### 2.2.1. Physical Appearance of the Construct

The THG we utilized in our study comprised N-isopropylacrylamide (NIPAAM), poly(ethylene glycol) diacrylate (PEGDA, Mn 575), 50-kDa hyaluronic acid (HA), ammonium persulfate, and tetramethylethylenediamine (TEMED). Several critical characteristics of THG material have been extensively documented in the literature, including its low critical solution temperature, swelling ratio, rheological properties, in vitro degradation, electron microscopy-based morphology, and the in vitro release kinetics of bevacizumab [23,30,31]. Consequently, to avoid redundancy, this section solely focuses on selected ACA-relevant aspects of this material.

**Figure 1:**
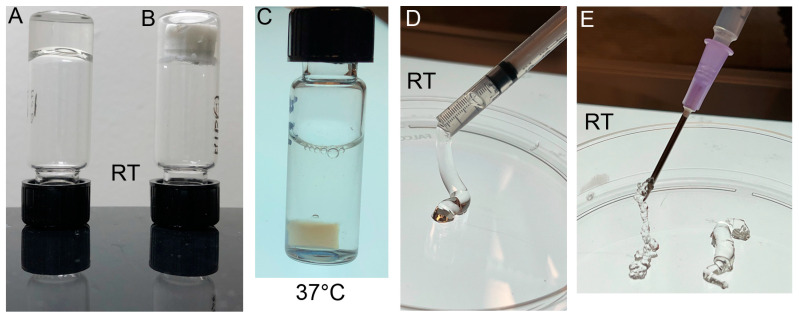
Appearances of polymerized THG. (**A**) Polymerized THG at room temperature (RT). (**B**) Lyophilized THG. (**C**) Collapsed THG (depicted in panel (**A**)) after being placed at 37 °C. (**D**) The THG formed in a syringe for injection into target sites. The syringe was cut to illustrate the consistency of the THG at RT. (**E**) The ACA-containing THG can be injected via a needle at a temperature < 32 °C.

Consistent with earlier studies, our results demonstrated that, at room temperature (RT), the ACA–THG construct has an expected transparent gel structure and is readily injectable using a syringe (Figure 1) [23]. As intended, when placed at 37 °C, the construct contracts, trapping the ACA into the THG network and becoming opaque (Figure 1C).

#### 2.2.2. Fourier Transform Infrared (FTIR) Spectroscopy of the ACA–THG

**Figure 2:**
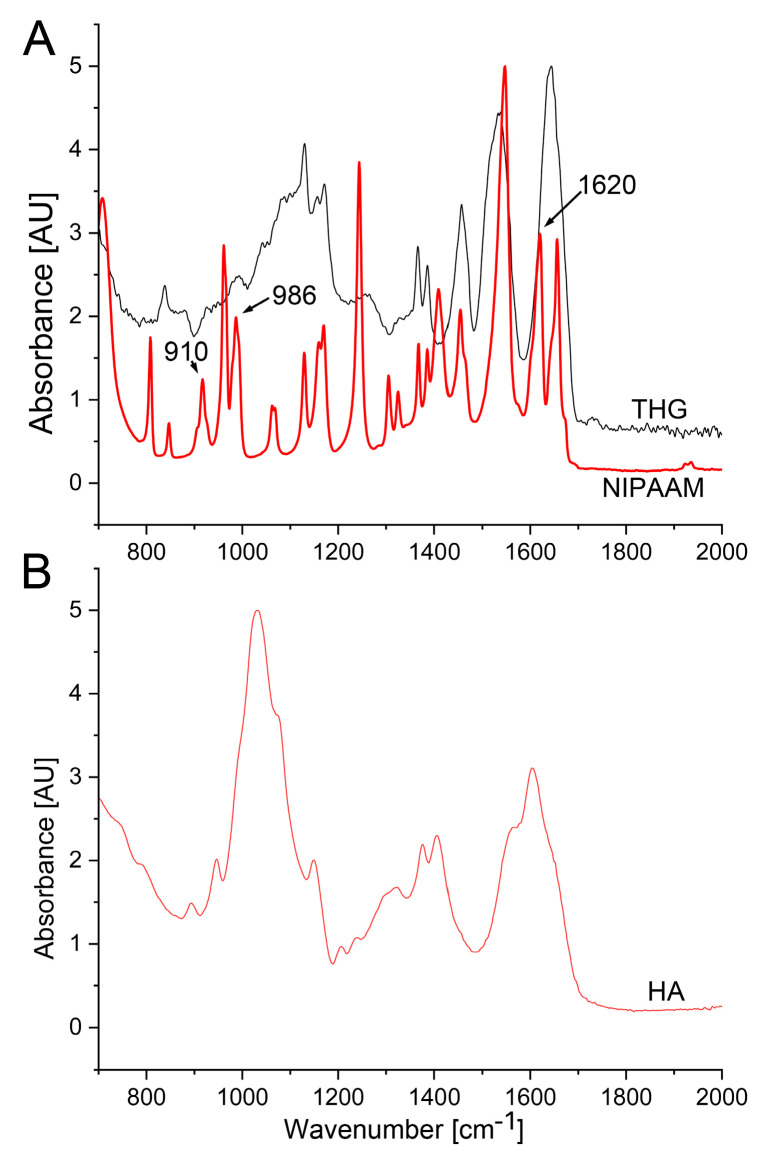
FTIR spectra of THG, monomeric NIPAAM (**A**), and HA (**B**). Methylene and vinyl peaks (arrows) present in NIPAAm at 1620 cm^−1^, 986 cm^−1^, and 910 cm ^-1^, respectively, disappeared in THG, signifying the formation of single-bond C–H peaks in PEGDA-crosslinked NIPAAM-HA hydrogels.

We analyzed the THG material (polymer) in comparison to its essential monomers, 50–70 kDa HA, and NIPAAM, which were used to create the hydrogel. The FTIR spectra (Figure 2) align with those reported by Egbu et al. [23]. These researchers, who developed and analyzed the identical THG construct, determined that the NIPAAM spectra exhibit characteristic methylene and vinyl peaks at approximately 1620 cm^−1^, 986 cm^−1^, and 910 cm^−1^, which vanish in the THG spectra (Figure 2A, marked by arrows). This observation indicates the formation of single-bond C–H peaks in the poly(ethylene glycol) diacrylate (PEGDA)-crosslinked NIPAAM-HA THG.

Here, by synthesizing the same material, we confirmed these observations (Figure 2A). Moreover, the NIPAAM (Figure 2A) and HA (Figure 2B) spectra of the materials we utilized here matched the spectra of the corresponding materials from the database included in the Spectrum software (version 10.5.4.738, Perkin Elmer Inc., Waltham, MA, USA). The search score for HA was 0.73, and for NIPAAM, the search score was 0.86. These high scores further confirm the proper spectral characteristics of the materials employed here.

### 2.3. Assays of the ACA Released from the ACA–THG Constructs

The collagen-rich matrix in mature scars exhibits remarkable stability and is not dissolvable using pharmacological methods. In orthopedic practice, bacterial collagenase may be applied to digest fibrotic cords that form in patients with Dupuytren’s contracture [32,33]. A similar approach has also been approved for penile fibrosis in patients with Peyronie’s disease [34].

While bacterial collagenases are potent enzymes for breaking down collagen-rich scar tissue, their application near various tissues, such as cartilage and peripheral nerves, carries a substantial risk of causing irreversible collagenolytic damage [32]. Consequently, in the context of posttraumatic fibrosis, a more suitable approach to prevent excessive scarring is to reduce the formation of scar tissue in the early stages of the healing process. Also, because scarring of orthopedically relevant tissues is a long-term process, a successful antifibrotic agent should be applied continuously to achieve satisfactory outcomes [35].

**Figure 3:**
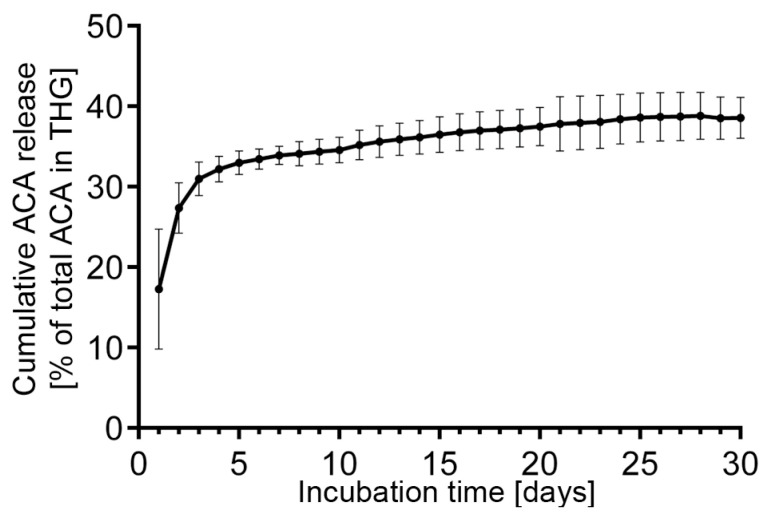
In vitro release profiles of the ACA from the THG construct in PBS (pH 7.4) at 37 °C.

Measurements of ACA release showed that, on average, approximately 16% of the ACA content present in the ACA–THG discs was released within the first day of incubation at 37 °C (Figure 3). Subsequently, about 30% of the total ACA is released in the initial 10 days of incubation. Following this, the rate of ACA release decelerates. After 30 days of incubation, roughly 32% of the initial ACA content is released from the ACA–THG disk (Figure 3).

We propose that the initial, more rapid release of ACA is primarily attributed to the initial shrinkage of THG when introduced to a 37 °C environment. This relatively fast initial release is expected to enhance the therapeutic potential of ACA by providing an “initial loading” of target sites with the antibody.

The above release profile demonstrates favorable ACA delivery characteristics. Within the 1 mL ACA–THG disks, the total ACA amount was 30 mg. After 30 days, approximately 10 mg of the antibody (32%) was released. By extrapolating these results and considering that we can apply at least 5 mL of ACA–THG in a few injection spots to the rabbits’ injury areas, we can potentially deliver 10 mg × 5 = 50 mg of ACA per 30 days, using conditions that we have tested in vitro. This amount is 1.7 times higher than the highest dose (30 mg/month) and 25 times higher than the lowest dose (2 mg/month) that we have effectively administered in vivo in a rabbit-based arthrofibrosis model using a pump-based delivery system [21].

The highly modular design of the ACA–THG system, which allows for adjusting the amount of ACA in the hydrogel material and the ACA–THG volume, will enable us to tailor the optimal delivery parameters to achieve the most effective therapeutic outcomes.

### 2.4. Analysis of ACA’s Structural Integrity

**Figure 4:**
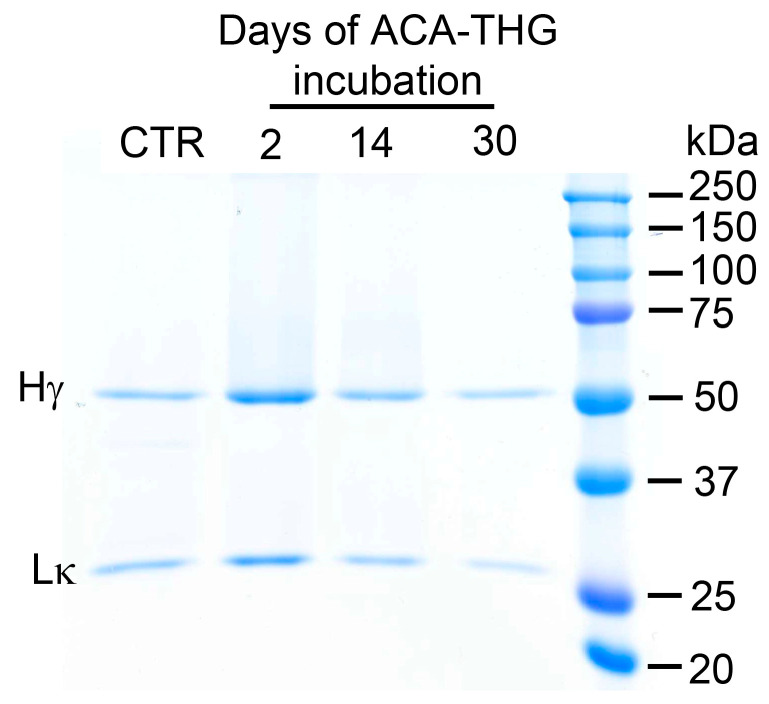
Electrophoretic analysis displaying the structural integrity of ACA released from the ACA–THG construct. The ACA released from the construct on days 2, 14, and 30 is highlighted. Control ACA (CTR) utilized in the construction process and molecular mass markers are also included. The gel demonstrates intact heavy (Hγ) and light (Lκ) ACA chains.

In our previous studies, we confirmed the stability of ACA at 37 °C in vitro and when present in subcutaneous pumps implanted in rabbits for a minimum of 30 days [21]. Similarly, the ACA released from the ACA–THG discs (Figure 1C) exhibited structural integrity, as evidenced by the antibody’s intact heavy and light chains (Figure 4).

Many therapeutic antibodies are prone to undesirable aggregation, which can occur at various stages of antibody preparation, such as purification, concentration, or storage [36]. It is crucial to emphasize that the therapeutic efficacy of these antibodies diminishes when they aggregate. Given the potential for aggregation, we investigated whether the monomeric form of ACA, employed in creating ACA–THG constructs, remained unaltered after being released from the hydrogel discs.

Electrophoresis under reducing and denaturing conditions revealed the correct monomeric structure of the heavy and light ACA chains (see Figure 4). Nevertheless, to thoroughly assess the aggregation status of ACA in its native conformation, further analysis using size exclusion chromatography (SEC) was required.

**Figure 5:**
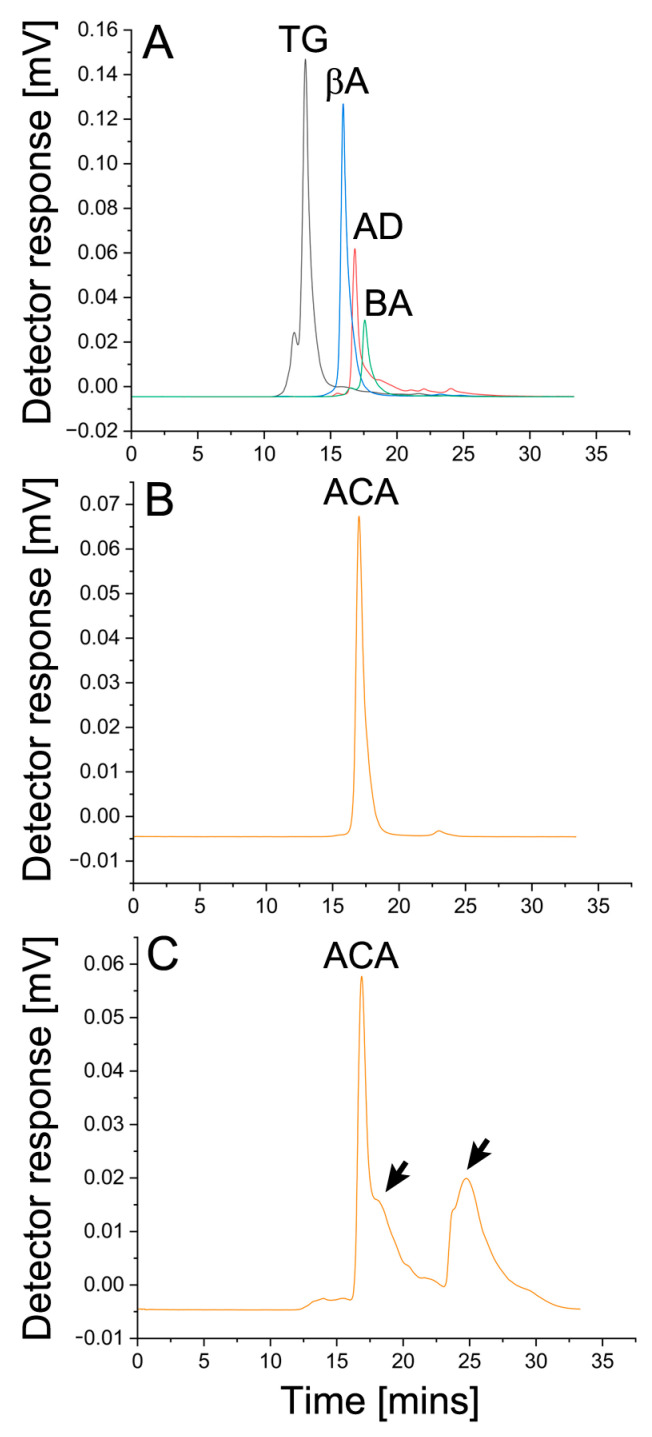
HPLC SEC analysis of ACA release from the ACA–THG construct. (**A**), Chromatogram depicting elution profiles of the 669 kDa (TG, thyroglobulin), 200 kDa (βA, β-amylase), 150 kDa (AD, alcohol dehydrogenase), and 66 kDa (BA, bovine albumin) markers. (**B**), ACA elution profile. (**C**), Chromatogram illustrating ACA, estimated to have a molecular mass of approximately 150 kDa, released from the hydrogel material after a 30-day incubation period. The 150 kDa peak represents ACA; arrows indicate additional peaks of lower molecular masses.

The SEC elution profiles clearly show that the retention time of ACA released from the ACA–THG material after 30 days of incubation at 37 °C closely resembled that of the 150-kDa molecular mass marker (Figure 5).

Because we did not observe any protein peaks with shorter retention times, which would typically indicate the formation of ACA oligomers, the SEC-based assays indicated that within the experimental conditions of our study, ACA retained its native monomeric structure without any signs of aggregation.

Still, we observed a broad peak of low-molecular-mass molecules. Considering that the electrophoretic assays did not show ACA degradation, we suggest that this peak represents HA fragments or other THG elements released due to hydrogel degradation.

### 2.5. Antigen-Binding Characteristics

**Figure 6:**
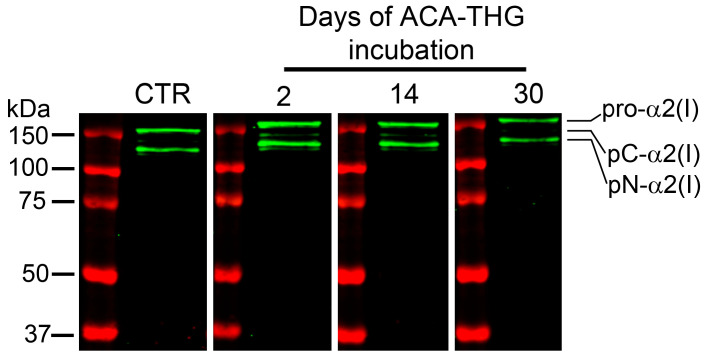
Analysis of target-binding properties of ACA released from the ACA–THG construct on days 2, 14, and 30. Results for control ACA (CTR, ACA used to fabricate the THG construct) are also shown. Intact pro-α2(I) chains and partially processed pN-α2(I) chains (both include the ACA-binding target) are indicated. Please note that procollagen I samples, each accompanied by the molecular mass markers, were run on a 7.5% gel, and then separated proteins were transferred onto a nitrocellulose membrane. The membrane was cut into four strips along the marker lanes. Subsequently, each strip was probed with indicated ACA variants.

While examining its structural integrity and aggregation status, we also investigated the ability of ACA released from the ACA–THG construct to interact with its native target, i.e., α2(I)Ct. To assess this, we conducted Western blot experiments using human procollagen I as the specific target and the released ACA as the primary antibody. The binding of ACA to the procollagen I target was visualized using goat anti-human IgG conjugated with a fluorophore (Figure 6).

The observed ACA target binding demonstrates that none of the components of THG interfered with the antigen-binding domains of ACA. This result indicates that ACA retained its target-specific binding properties on days 2, 14, and 30 after release. The specificity of antigen binding is evident from the fact that ACA exclusively recognized the intact pro-α2(I) chain and its partially degraded forms, namely pC-α2(I) (pro-α2(I) chain degraded from the N terminus) and pN-α2(I) (pro-α2(I) chain degraded from the C terminus). Typically found in dermal fibroblast culture media, these forms include the α2(I)Ct epitope. In contrast, ACA did not recognize any pro-α1(I) chain variants lacking the α2(I)Ct target. This further underscores the antigen specificity of this antibody incorporated into the THG architecture for a minimum of 30 days.

### 2.6. Safety of the ACA–THG Constructs

Previous studies of the THG material, similar to that used in our study, demonstrated its safety [24,26,28,37]. Here, we assessed the safety of the THG construct in the context of neurons, as we aimed to employ it as a delivery platform for ACA in orthopedically relevant peripheral nerve injury sites. Our primary concern was whether the construct might adversely affect the viability and growth of neurons. This concern was of particular importance, given that the regeneration of injured peripheral nerves relies on the timely growth of axons from the proximal to the distal stump of the injured nerve.

**Figure 7:**
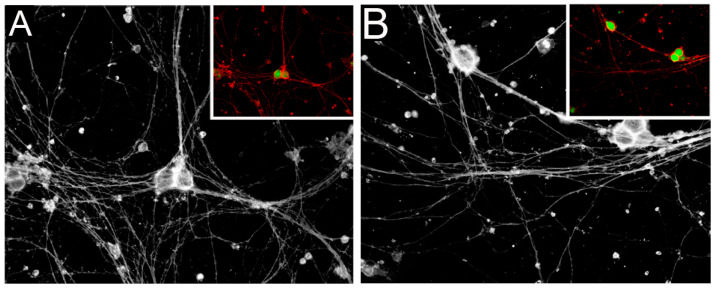
Comparison of cell viability and neurite outgrowth in dorsal root ganglion neurons in two conditions: without (**A**) and with (**B**) the ACA and THG constructs. In condition B, the cell culture contained ACA, while the THG disc was situated in a transwell insert submerged in the media. After 7 days, neuron viability was assessed using a cell-permeable indicator that emits green fluorescence within living cells. Simultaneously, neurite outgrowth was evaluated by staining the outer cell membranes in bright red/orange. Notably, the larger images are presented in black and white to enhance contrast for better visualization of delicate axons. Additionally, corresponding inserts display markers for neuron viability (green) and proliferation (orange/red) using fluorescence.

Our research focused on investigating the impact of ACA–THG components on cultured neurons. To assess cell viability, we employed a cell-permeable viability marker. When processed by live cells, this marker emits green fluorescence. Simultaneously, we utilized a bright orange-red stain that adhered to the outer cell membranes, facilitating the monitoring of neurite outgrowth. Figure 7 illustrates a typical dual-staining pattern of the analyzed neurons.

To quantify neural outgrowth, we measured the surface area occupied by the axons within the entire photographed area using a 20× objective. Our results indicate that there were no discernible differences among the areas measured for all analyzed groups (Table 1).

We also measured the percentage of viable cells, characterized by green fluorescence, visible in the photographed areas. Our assays determined that in all analyzed groups 100% cells were viable.

These results corroborate our earlier research showing that the continuous application of ACA into injured joints does not impact peripheral nerves, as evident by the proper histological features of the analyzed sciatic nerves [21]. Together, these results provided the necessary assurance for proceeding with in vivo tests, as discussed below.

### 2.7. Nerve Injury Model

In a previous study, we established rabbit-based models for sciatic nerve crash injury, partial transection, and segmental injury to investigate neuronal scarring [38]. In this study, we employed a partial transection nerve injury model to assess the THG-based ACA delivery system.

**Figure 8:**
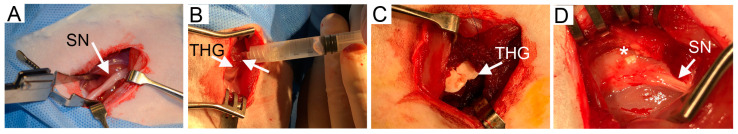
Selected steps in a nerve injury model and a THG-based ACA delivery into injury sites. (**A**), Exposing the sciatic nerve (SN) to perform surgical injury. (**B**), Applying the ACA–THG material. (**C**), Contraction of the thermoresponsive construct at body temperature. (**D**), The site of the ACA–THG injection 8 weeks post-surgery.

The application of the ACA–THG construct was uncomplicated (Figure 8). Once the construct reached the nerve injury sites, it underwent contraction in response to body temperature (as depicted in Figure 8C). Upon examining the injection site 8 weeks after surgery, a small fragment resembling the original THG material was discovered in one of the rabbits (Figure 8D, marked by an asterisk). No such fragments were observed in the remaining rabbits. These observations indicate that the ACA–THG constructs underwent degradation in vivo within the 8-week experimental period.

We concluded that the ACA–THG material did not lead to any macroscopic abnormalities in the adjacent muscle and nerve tissues (Figure 8D). This observation aligns with the findings of d’Este et al., who demonstrated that a similar thermoresponsive hydrogel, when applied in a rabbit model with an osteochondral defect, did not induce any microscopic pathological effects. They noted the absence of abnormalities in distant tissues and organs and soft tissues surrounding the injured knee joint [24].

### 2.8. Detecting Humanized ACA in Rabbit Sera and Evaluating Its Antigen-Binding Activity

**Figure 9:**
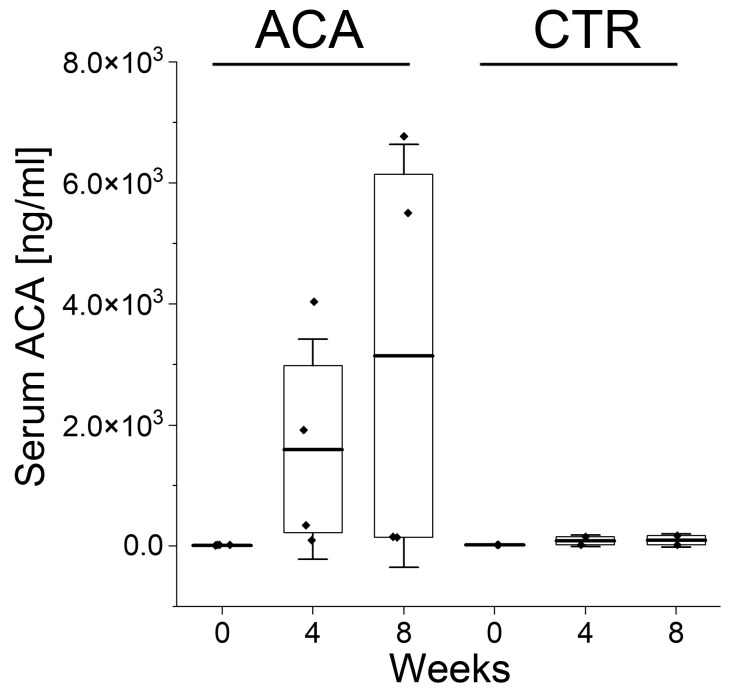
Graphic representation of the ACA concentrations in the rabbits’ sera 4 and 8 weeks after injecting the ACA–THG constructs into injury sites. Results are also presented for the control group (CTR) receiving the THG construct without ACA. The interquartile range between the 25th and 75th percentiles determines each box. The lines within the boxes represent the means, while the whiskers delineate the SD values. The black diamonds represent individual ACA concentration values.

Because the ACA delivered by THG reaches both the nerve injury sites and the bloodstream, we used a human κ chain-specific enzyme-linked immunosorbent assay (ELISA) to determine that 4 weeks after implanting the ACA–THG construct, the serum concentration of this antibody in rabbits averaged 1.5 μg/mL. After 8 weeks, it increased to 3.1 μg/mL (Figure 9). In contrast, the control group that received the THG material only displayed ELISA signals at background levels (Figure 9).

While the number of rabbits in our exploratory study was limited in both the treated and control groups, preventing us from conducting formal statistical analyses on time-dependent changes in ACA serum concentration, these pilot studies suggest that the antibody was continuously released from the time of ACA–THG construct injection during the 8-week experimental period. However, it is essential to note that the half-life of ACA in rabbit sera is a parameter that we have not yet investigated. As this parameter impacts the calculation of continuous ACA release dynamics, future studies will be necessary to address the half-life issue to determine serum ACA concentration profiles more precisely.

In our in vivo experiment, we aimed to address a crucial question: whether ACA released into the bloodstream over 8 weeks maintains its antigen-binding activity. We administered the ACA construct to four rabbits. We successfully collected enough serum from two of them, allowing us to measure the serum concentration of this antibody and assess its antigen-binding activity in triplicate. Simultaneously, we collected sufficient serum from both rabbits in the control group for comparison.

The sera were used in ELISA-based ACA-procollagen I binding experiments. They revealed that the average absorbance of the ELISA reaction samples in the ACA-treated group was 0.1 (SD = 0.06). In contrast, the absorbance remained at the background level in the control group, which was equivalent to the negative control. Based on these results, we concluded that the ACA in the rabbit sera collected 30 days after nerve injury retained its antigen-binding activity.

Due to the limited sample size, we refrained from attempting to quantify the amount of procollagen I-bound ACA. Instead, we treated these results in a binary manner: either we observed antigen binding, or we did not.

### 2.9. CMAP Recordings

**Figure 10:**
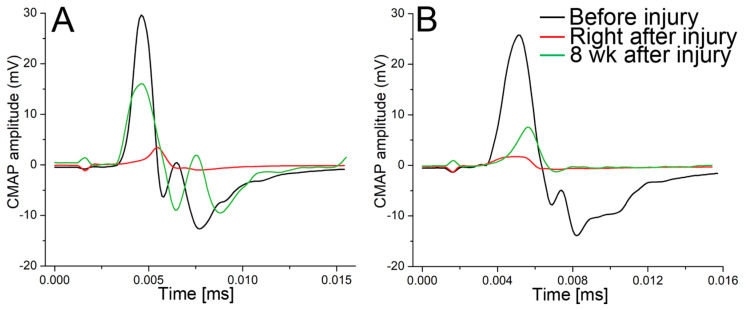
Pilot measurements of the CMAP in a rabbit treated with ACA–THG (**A**) and untreated (**B**) control. The sciatic nerves of the rabbits were partially transected. The CMAP was measured before, right after, and 8 weeks after injury.

We conducted CMAP recordings at three key timepoints: before the injury, immediately after the injury, and 8 weeks following the injury to the sciatic nerve. For the rabbits treated with ACA, the average maximal CMAPs and their corresponding standard deviations (SD) at these timepoints were as follows: 28.2 mV (SD = 6.8), 11.1 mV (SD = 7.8), and 13.8 mV (SD = 8.6). In the control group, the corresponding values were 25.7 mV (SD = 0.2), 12.2 mV (SD = 14.5), and 12.7 mV (SD = 7.0) (Figure 10).

Notably, one rabbit from the ACA-treated group was excluded from the calculations because the CMAP values were identical before and immediately after the injury. In this case, we expect that we did not create significant nerve damage due to a surgical error.

The results above reveal that the CMAP averages in the ACA-treated and control groups were similar 8 weeks after nerve injury. This observation suggests that ACA treatment did not enhance the recovery kinetics of nerve function. Nevertheless, it is crucial to consider that our 8-week experimental period might have been insufficient to predict a complete recovery of nerve function. According to Schmitz and Beer, the earliest point we can expect recovery from a similar nerve injury in rabbits is 9 weeks post-injury [39]. Therefore, a significantly extended recovery period may be required to determine the impact of ACA on CMAP recovery in injured nerves.

Furthermore, because our main objective was to assess the efficacy of THG in delivering ACA, we did not examine how this antibody might affect scar tissue formation at nerve injury sites. Given the established utility of THG constructs in effectively delivering ACA without modifying its crucial properties, further investigations are necessary. In peripheral nerve regeneration, these studies should emphasize ACA dosage and its impact on neural scar formation, nerve function recovery, and quantitative and qualitative factors describing regenerating axons.

### 2.10. Potential Clinical Impact

#### 2.10.1. Short-Term Impact

The current emphasis on preventive medicine extends to decreasing the excessive formation of fibrotic scars in musculoskeletal tissues. Our current inability to prevent unwanted scar formation after an injury leads to prolonged disability and failure to perform at preinjury activity levels. In the short term, implementing the ACA–THG-based method of fibrosis prevention after injury should allow for an earlier and more reliable return to functional activities. Injured patients may return to work sooner after sustaining an injury and may not need to change their responsibilities to accommodate the consequences of an injury.

#### 2.10.2. Long-Term Impact

The long-term promise of modulating the scar formation pattern and decreasing posttraumatic fibrosis will improve the healing potential of injured tissues and potentially save limbs that are unsalvageable with our current methods in clinical medicine. In the absence of excessive scar tissue formation, posttraumatic joint contractures should decrease, and injured tissues should better heal in a manner that minimizes the effects of trauma.

If excessive scarring is prevented, individuals recovering from traumatic orthopedic injuries should have higher patient satisfaction and improved functionality with subsequent increased work longevity. The clinical application of the proposed treatment may revolutionize fibrosis management after orthopedic injuries.

## 3. Conclusions

A controlled delivery system for ACA employing a thermoresponsive hydrogel was successfully implemented in a rabbit model of peripheral nerve injury. The ACA–THG construct exhibited a slow release of ACA with no notable burst release features. Hydrogel-encapsulated ACA maintained its antigen-binding properties upon release, both in vitro and in vivo. Notably, the construct demonstrated no adverse effects when implanted around the sites of peripheral nerve injury.

The results presented herein suggest that the THG-based ACA delivery platform holds promise for the controlled release of therapeutic antibodies targeting the soft components of orthopedically relevant tissues. Further long-term studies will be crucial in determining the efficacy of this material in limiting fibrotic scarring in tendons, ligaments, muscles, joint capsules, and peripheral nerves.

## 4. Materials and Methods

### 4.1. Production of ACA

We employed a well-studied ACA developed by our research team [20,29]. The humanized ACA IgG4 variant was produced using FreeStyle CHO-S cells (ThermoFisher Scientific, Waltham, MA, USA) cultured in a bioreactor (BioFlo 115, Eppendorf, New Brunswick, NJ, USA) [21]. In accordance with our published protocols, we purified the antibody from cell culture media using protein-L-based affinity chromatography (GenScript Biotech, Piscataway, NJ, USA) [29].

### 4.2. Fabrication of the ACA–THG Construct

Our exploration of drug delivery for orthopedically relevant targets, such as bone, cartilage, muscle, and peripheral nerves, revealed various customized constructs made from synthetic and natural compounds that could be useful for this project [40,41,42]. After a review of the published literature, we selected a well-characterized thermosensitive hydrogel (THG) with a well-defined antibody-release profile and suitable physicochemical properties [23,27]. Additionally, our choice was influenced by reports of a similar material being effectively and safely applied in experiments to repair joint tissues in vivo [24,25].

The THG comprises N-isopropylacrylamide (40 mg/mL, NIPAAM, MilliporeSigma, Burlington, MA, USA), poly(ethylene glycol) diacrylate (2.5 μL/mL, PEGDA, Mn 575, MilliporeSigma), 50-kDa hyaluronic acid (10 mg/mL, HA, Matexcel, Shirley, NY, USA), ammonium persulfate (4 mg/mL, APS, ThermoFisher Scientific), and tetramethylethylenediamine (20 μL/mL, TEMED, ThermoFisher Scientific).

Initially, all components of THG, except TEMED and APS, were mixed at 4 °C for 24 h. After adding APS, the mixture was sterilized using a 0.22 μm filter. Filter-sterilized ACA was added to achieve a final 30 mg/mL concentration. Finally, TEMED was introduced to catalyze the polymerization of the THG construct.

The ACA–THG constructs were poured into glass vials during the liquid state for in vitro testing (see below). When prepared for in vivo studies in rabbits (see below), the liquid constructs were drawn into 3 mL syringes. These ACA–THG constructs were kept at 4 °C for 48 h to allow complete polymerization. It is important to note that the THG maintains an open structure within the temperature range of 4 °C to 30 °C. As the temperature increases (i.e., to body temperature), the THG’s structure contracts, effectively trapping the antibody within the interlacing polymer molecules [24,25,30,31,43,44].

### 4.3. Fourier Transform Infrared (FTIR) Spectroscopy

We employed an FTIR spectroscope (Spotlight 400, Perkin Elmer, Inc.) to examine the chemical characteristics of the THG material [23]. In brief, polymerized THG samples were freeze-dried and pressed against a high-refractive index prism. Absorption was measured with 8 scans at a resolution of 4 cm^−1^ for wavenumbers ranging from 700 to 4000 cm^−1^. The FTIR spectra were analyzed according to the previously described methods (Spectrum software version 10.5.4.738, Perkin Elmer, Inc.) [23].

### 4.4. ACA Release Studies

To examine the release of ACA, we utilized discs formed in glass vials from 1 mL of ACA–THG mixtures. The discs were immersed in phosphate-buffered saline (PBS) and placed in a cell culture incubator at 37 °C, where they contracted. Subsequently, we collected PBS every 24 h, recorded its volume, and refilled the vials with fresh PBS.

The samples collected during the 30 days were stored at −20 °C. After the experiment, we determined the amount of ACA released in each sample using a colorimetric method with an IgG-based standard curve (Bio-Rad Laboratories, Hercules, CA, USA).

The amount of ACA released was expressed as a percentage of the antibody content in the ACA–THG discs. We presented the kinetics of ACA release graphically in a time/released ACA percentage plot. Each data point on the plot represents the mean values obtained from three independent experiments, with SD values included.

### 4.5. Evaluating the Structural Integrity of Released ACA from the ACA–THG Construct

Considering that the integrity and activity of a biological substance, including a therapeutic antibody, stored within a biomaterial can change over time, we analyzed the ACA released from the THG construct at various timepoints using electrophoretic methods. The proper electrophoretic migration of intact ACA heavy and light chains indicated that the antibody maintained its integrity throughout the experiment.

In brief, we loaded an aliquot of a sample containing the ACA released after a specific incubation period onto a 10% polyacrylamide gel. After electrophoretic separation of the heavy and light chains, we visualized them by staining them with Coomassie blue dye (Bio-Rad Laboratories). For reference, we used molecular mass markers and the original ACA stock.

### 4.6. Checking the ACA Aggregation Status

As therapeutic antibodies are prone to unwanted aggregation, we utilized high-performance liquid chromatography (HPLC, Star chromatography workstation, VARIAN, Inc., Palo Alto, CA, USA) to assess the aggregation status of ACA after its 30-day incubation within the ACA–THG constructs. In brief, the protein samples under examination were injected into a size-exclusion column (SEC, BioBasic-SEC-300, ThermoFisher Scientific) preequilibrated with PBS. These samples were then eluted at a flow rate of 1 mL/min, and the protein peaks were detected using a UV detector (UV-VIS Detector Model 345, VARIAN, Inc.) set at 280 nm. We evaluated the original ACA and the ACA released from the ACA–THG discs. Additionally, we employed molecular mass protein markers spanning the mass range of 66 kDa to 200 kDa (from MilliporeSigma). SEC elution profiles were generated and scrutinized to determine the molecular mass of the ACA samples.

### 4.7. Assessing the Target-Binding Activity of ACA Released from the ACA–THG Construct In Vitro

We also investigated whether ACA, once released from the ACA–THG construct, maintains its ability to bind to its native target, the C-terminal telopeptide of the collagen I α2(I) chain (α2(I)Ct) [20]. To accomplish this, we conducted Western blot experiments using human procollagen I as the native ACA target. Procollagen I was isolated from human dermal fibroblast cultures following the methods described by Kadler et al. [45].

ACA released from the ACA–THG construct at various timepoints during the 30-day incubation period served as the primary antibody. The secondary antibody was goat antihuman IgG conjugated with an infrared fluorophore (IRDye 800CW, LI-COR Biosciences, Lincoln, NE, USA).

Western blot signals were observed using an Odyssey CLx imager (LI-COR Biosciences). Positive Western blot signals, which corresponded to various forms of the α2(I) chains, indicated that the released ACA maintained its target-specific binding activity.

### 4.8. Evaluation of ACA-TGH Constructs’ Safety in the Context of Neuron Growth

To assess the safety of the THG-based ACA delivery platform in the context of injured nerves, we analyzed the impact of the ACA–THG construct on neuron viability and growth. We also included a construct containing control human IgG (hIgG-THG), a THG construct with no antibody, and a control containing neither THG nor the antibodies (CTR).

We procured dorsal root ganglion rat neurons from Lonza Group AG (Basel, Switzerland). We cultured them in primary neuron growth medium (PNGM BulletKit, Lonza Group AG) with or without ACA–THG constructs in 24-well plates coated with poly-D-lysine/laminin (Amsbio LLC, Cambridge, MA, USA).

The hydrogel constructs were prepared within the transwell inserts as described above. After thorough polymerization, these inserts were placed into wells containing neurons. The cell cultures were kept for seven days.

Subsequently, we performed microscopic analysis to evaluate the viability and growth of the neurons. Neuron viability was assessed using a cell-permeable indicator that emits green fluorescence only in living cells. Simultaneously, neurite outgrowth was measured by staining the outer cell membranes with a bright red/orange fluorescent dye using the Neurite Outgrowth Staining Kit from ThermoFisher Scientific (Molecular Probes, Eugene, OR, USA). The observations of neurons were made under an inverted fluorescent light microscope (Eclipse Ti, Nikon Corporation, Tokyo, Japan) equipped with a digital camera (DS-Qi1Mc, Nikon) controlled by the NIS-Elements AR software, version 3.22.14 (Nikon).

To assess neurite outgrowth, we quantified the surface area occupied by neurites across the entire photographed field using a red fluorescence filter and a 20× objective. Simultaneously, we determined the percentage of viable cells by counting all visible cells within the entire photographed area, utilizing a green fluorescence filter and a 20× objective. For each analyzed group, three randomly selected areas were photographed. Subsequently, we computed the average area values for each group and presented them alongside the corresponding standard deviation. The microscopic measurements were conducted using the NIS-Elements AR software, version 3.22.14 from Nikon.

### 4.9. Rabbit Nerve Injury Model

The Thomas Jefferson Institutional Animal Care and Use Committee (IACUC) approved the animal studies described below. In these studies, we assessed the behavior of the ACA–THG construct in a rabbit nerve injury model. Building on our previous experience, we employed a partial transection nerve injury model [38].

In brief, after administering anesthesia, we prepared the incision site by shaving the surrounding area. Subsequently, we disinfected the skin using a povidone-iodine scrub from the sacrum level to the knee. Then, we washed the area with gauze soaked in 70% isopropyl alcohol. The operated rabbit was positioned laterally with the hind legs abducted and the knees flexed at a 90° angle. An incision over the lateral thigh provided access to the sciatic nerve. This incision was centered over the palpable raphe on the posterolateral aspect of the hind leg, between the greater trochanter and the lateral tubercle of the knee. After making a sharp incision through the skin and subcutaneous fascia, a thin fascia layer was revealed overlying the muscle. Cutting through this fascia allowed for a muscle-sparing approach to the posterior femur. The muscular septum between the semitendinosus and caput pelvinum bicipitis femoris muscle was identified and gently dissected.

The sciatic nerve, easily distinguishable within this interval, lay posteromedial and deep to the abductor cruris caudalis, with the adductor femoris proximally and the semimembranosus muscle distally. A delicate membrane covering and anchoring the sciatic nerve in place was cautiously opened, providing circumferential access to the nerve. The nerve was then manipulated and partially transected to create the injury model, as described in our previous work [19].

#### Administration of ACA–THG Constructs

Following the creation of nerve injuries, the syringes containing the polymerized ACA–THG material were retrieved from the refrigerator. Subsequently, 3 mL of the material was injected into the injury sites. The incisions were then sutured, and the rabbits were allowed to recover from anesthesia, resuming normal, unrestricted activities for 8 weeks. At the end of these 8 weeks, the rabbits were euthanized. Subsequently, the injury sites were examined for macroscopic signs of potential side effects caused by the ACA–THG material.

In our initial investigations, we administered ACA–THG constructs to four rabbits, two males and two females. In contrast, one male and one female rabbit received only THG.

### 4.10. Analyzing ACA in Rabbits’ Sera

#### 4.10.1. ACA Concentration Assays

Because ACA released from THG not only diffuses into nerve injury sites but also enters the bloodstream, we analyzed the serum concentration of this antibody before its application and again at 4 and 8 weeks after ACA application.

Given that ACA belongs to the human IgG4 isotype with a light κ chain, we utilized a human κ chain-specific ELISA (Abcam, Waltham, MA, USA). The test was performed following the manufacturer’s protocol, and the ACA concentration was determined based on the standard curve using a company-provided marker. Sera collected from the control group of rabbits were also analyzed.

#### 4.10.2. Antigen Binding of ACA in Rabbit Sera

In week 8, we assessed the antigen-binding activity of the ACA released from the ACA–THG constructs into the bloodstream. To accomplish this, we immobilized human procollagen I, a native ACA target, in the wells of a 96-well plate (Nunc MaxiSorp, ThermoFisher Scientific). We then blocked nonspecific binding sites by applying a 0.25% solution of bovine serum albumin (BSA, MilliporeSigma) dissolved in PBS with 0.05% Tween 20 (PBST, MilliporeSigma). Subsequently, the wells were thoroughly washed with PBST to eliminate unbound proteins.

Sera samples were diluted 1:1 with PBST, and 0.1 mL samples were added to the procollagen I-coated wells. After overnight incubation at 4 °C, we washed the wells with PBST. Then, we introduced a goat anti-human IgG biotin-conjugated antibody to the wells at a 1:20,000 dilution (MilliporeSigma). Following a 1 h incubation at room temperature, we rewashed the wells with PBST. Subsequently, we added streptavidin conjugated with alkaline phosphatase (AP) to the wells for 1 h (MilliporeSigma).

Finally, the wells were washed with PBST and carbonate buffer (MilliporeSigma). We detected the presence of procollagen–ACA complexes by adding the AP substrate, p-nitrophenyl phosphate (PNPP, MilliporeSigma). AP activity was neutralized by adding 25 µL of 0.5 N NaOH per well. We measured the absorbance of the developed color at 405 nm using a Benchmark Microplate Reader (Bio-Rad Laboratories). Data from analyzed samples prepared in triplicate were averaged.

Negative controls involved BSA-blocked wells with no procollagen I; positive controls consisted of wells coated with ACA.

### 4.11. Recording Compound Muscle Action Potentials (CMAP)

A neuroscientist (LC), unaware of the treatment conditions, conducted CMAP measurements at three specific timepoints: before nerve injury, immediately after the injury, and 8 weeks post-injury, just before the rabbits were euthanized.

The rabbits were anesthetized and placed on a heated pad. After exposing the sciatic nerve, a pair of needle electrodes used for stimulation was carefully inserted in parallel. They ran close to the sciatic nerve trunk, with a fixed distance of 0.5 cm between them. Recording electrodes were positioned within the gastrocnemius muscle, and reference electrodes were placed between the fourth and fifth digits of the animal’s foot. A grounding needle electrode was subcutaneously inserted into the animal’s tail.

We administered supramaximal stimuli lasting 0.5 milliseconds using a PowerLab 8/30 stimulator, and the CMAPs were recorded using a BioAMP amplifier (ADInstruments, Colorado Springs, CO, USA).

The CMAPs were amplified and filtered using a band-pass filter set between 50 and 3000 Hz. We employed Scope v.3.5.6 software (ADInstruments Inc.) to record and analyze the amplitudes of the CMAPs.

### 4.12. Data Analysis

The results of our studies are presented in the form of descriptive statistics. The described measurements are presented as means and corresponding standard deviations. Where appropriate, data are also presented as bar graphs (OriginPro 2023b, version 10.0.0.5.153, OriginLab Corporation, Northampton, MA, USA). The interquartile range between the 25th and 75th percentiles determined each bar. The lines within the bars represent the means, while the whiskers delineate the SD values.

## Figures and Tables

**Table 1:** Analysis of neurite outgrowth expressed as the surface area occupied by growing axons.

	Analyzed Group			
	ACA–THG	hIgG-THG	THG	CTR ^a^
Area occupied by the axons [µm^2^] (±SD)	37.9 × 10^3^ (16.9)	37.7 × 10^3^ (12.7)	34.6 × 10^3^ (18.6)	39.1 × 10^3^ (9.0)

^a^ A control group cultured neither in the antibody nor the THG presence.

## Data Availability

The data presented in this study are openly available in article.

## References

[1] Usher K.M., Zhu S., Mavropalias G., Carrino J.A., Zhao J., Xu J. (2019). Pathological mechanisms and therapeutic outlooks for arthrofibrosis. Bone Res..

[2] Cheuy V.A., Foran J.R.H., Paxton R.J., Bade M.J., Zeni J.A., Stevens-Lapsley J.E. (2017). Arthrofibrosis Associated with Total Knee Arthroplasty. J. Arthroplast..

[3] Kurtz S., Ong K., Lau E., Mowat F., Halpern M. (2007). Projections of primary and revision hip and knee arthroplasty in the United States from 2005 to 2030. J. Bone Jt. Surg. Am..

[4] Sharma V., Maheshwari A.V., Tsailas P.G., Ranawat A.S., Ranawat C.S. (2008). The results of knee manipulation for stiffness after total knee arthroplasty with or without an intra-articular steroid injection. Indian J. Orthop..

[5] Duplay E. (1872). Dr la peri-arthrite scapulo humerale et des raideurs de l’epaule qui en son la consequence. Arch. Gen. Med..

[6] Jones N.F., Ahn H.C., Eo S. (2012). Revision surgery for persistent and recurrent carpal tunnel syndrome and for failed carpal tunnel release. Plast. Reconstr. Surg..

[7] Amadio P.C. (2009). Interventions for recurrent/persistent carpal tunnel syndrome after carpal tunnel release. J. Hand. Surg. Am..

[8] Lowe J.B., Maggi S.P., Mackinnon S.E. (2004). The position of crossing branches of the medial antebrachial cutaneous nerve during cubital tunnel surgery in humans. Plast. Reconstr. Surg..

[9] Wang M.L., Rivlin M., Graham J.G., Beredjiklian P.K. (2018). Peripheral nerve injury, scarring, and recovery. Connect Tissue Res..

[10] Huckhagel T., Nuchtern J., Regelsberger J., Gelderblom M., Lefering R., TraumaRegister D.G.U. (2018). Nerve trauma of the lower extremity: Evaluation of 60,422 leg injured patients from the TraumaRegister DGU(R) between 2002 and 2015. Scand. J. Trauma Resusc. Emerg. Med..

[11] Scholz T., Krichevsky A., Sumarto A., Jaffurs D., Wirth G.A., Paydar K., Evans G.R. (2009). Peripheral nerve injuries: An international survey of current treatments and future perspectives. J. Reconstr. Microsurg..

[12] Karsy M., Watkins R., Jensen M.R., Guan J., Brock A.A., Mahan M.A. (2019). Trends and Cost Analysis of Upper Extremity Nerve Injury Using the National (Nationwide) Inpatient Sample. World Neurosurg..

[13] White E.S., Mantovani A.R. (2013). Inflammation, wound repair, and fibrosis: Reassessing the spectrum of tissue injury and resolution. J. Pathol..

[14] Schuster R., Rockel J.S., Kapoor M., Hinz B. (2021). The inflammatory speech of fibroblasts. Immunol. Rev..

[15] Hildebrand K.A., Schneider P.S., Mohtadi N.G.H., Ademola A., White N.J., Garven A., Walker R.E.A., Sajobi T.T., PERK 1 Investigators (2020). PrEvention of Posttraumatic contractuRes with Ketotifen 1 (PERK 1): A Randomized Clinical Trial. J. Orthop. Trauma.

[16] Nath R.K., Kwon B., Mackinnon S.E., Jensen J.N., Reznik S., Boutros S. (1998). Antibody to transforming growth factor beta reduces collagen production in injured peripheral nerve. Plast. Reconstr. Surg..

[17] Xue J.W., Jiao J.B., Liu X.F., Jiang Y.T., Yang G., Li C.Y., Yin W.T., Ling L. (2016). Inhibition of Peripheral Nerve Scarring by Calcium Antagonists, Also Known as Calcium Channel Blockers. Artif. Organs..

[18] Steplewski A., Fertala J., Beredjiklian P.K., Abboud J.A., Wang M.L., Namdari S., Barlow J., Rivlin M., Arnold W.V., Kostas J. (2016). Auxiliary proteins that facilitate formation of collagen-rich deposits in the posterior knee capsule in a rabbit-based joint contracture model. J. Orthop. Res..

[19] Rivlin M., Miller A., Tulipan J., Beredjiklian P.K., Wang M.L., Fertala J., Steplewski A., Kostas J., Fertala A. (2017). Patterns of production of collagen-rich deposits in peripheral nerves in response to injury: A pilot study in a rabbit model. Brain Behav..

[20] Chung H.J., Steplewski A., Chung K.Y., Uitto J., Fertala A. (2008). Collagen fibril formation. A new target to limit fibrosis. J. Biol. Chem..

[21] Steplewski A., Fertala J., Tomlinson R.E., Wang M.L., Donahue A., Arnold W.V., Rivlin M., Beredjiklian P.K., Abboud J.A., Namdari S. (2021). Mechanisms of reducing joint stiffness by blocking collagen fibrillogenesis in a rabbit model of posttraumatic arthrofibrosis. PLoS ONE.

[22] Grainger D.W. (2004). Controlled-release and local delivery of therapeutic antibodies. Expert Opin. Biol. Ther..

[23] Egbu R., Brocchini S., Khaw P.T., Awwad S. (2018). Antibody loaded collapsible hyaluronic acid hydrogels for intraocular delivery. Eur. J. Pharm. Biopharm..

[24] D’Este M., Sprecher C.M., Milz S., Nehrbass D., Dresing I., Zeiter S., Alini M., Eglin D. (2016). Evaluation of an injectable thermoresponsive hyaluronan hydrogel in a rabbit osteochondral defect model. J. Biomed. Mater. Res. A.

[25] Ter Boo G.J., Schmid T., Zderic I., Nehrbass D., Camenisch K., Richards R.G., Grijpma D.W., Moriarty T.F., Eglin D. (2018). Local application of a gentamicin-loaded thermo-responsive hydrogel allows for fracture healing upon clearance of a high Staphylococcus aureus load in a rabbit model. Eur. Cell Mater..

[26] Kim S., Kang-Mieler J.J., Liu W., Wang Z., Yiu G., Teixeira L.B., Mieler W.F., Thomasy S.M. (2020). Safety and biocompatibility of aflibercept-loaded microsphere thermo-responsive hydrogel drug delivery system in a nonhuman primate model. Transl. Vis. Sci. Technol..

[27] Jacob S., Nair A.B., Shah J., Sreeharsha N., Gupta S., Shinu P. (2021). Emerging Role of Hydrogels in Drug Delivery Systems, Tissue Engineering and Wound Management. Pharmaceutics.

[28] Li H., Sun H., Liu Y., Yuan B., Hu J., Jiang Y., Li Q., Cao S., Liu H., Xiao B. (2023). Toxicology and safety research of poly (N-isopropylacrylamide)-based thermosensitive nanogels. Environ. Sci. Nano.

[29] Fertala J., Steplewski A., Kostas J., Beredjiklian P., Williams G., Arnold W., Abboud J., Bhardwaj A., Hou C., Fertala A. (2013). Engineering and characterization of the chimeric antibody that targets the C-terminal telopeptide of the alpha2 chain of human collagen I: A next step in the quest to reduce localized fibrosis. Connect Tissue Res..

[30] Awwad S., Abubakre A., Angkawinitwong U., Khaw P.T., Brocchini S. (2019). In situ antibody-loaded hydrogel for intravitreal delivery. Eur. J. Pharm. Sci..

[31] Awwad S., Al-Shohani A., Khaw P.T., Brocchini S. (2018). Comparative Study of In Situ Loaded Antibody and PEG-Fab NIPAAM Gels. Macromol. Biosci..

[32] Cooper T.B., Poonit K., Yao C., Jin Z., Zheng J., Yan H. (2020). The efficacies and limitations of fasciectomy and collagenase clostridium histolyticum in Dupuytren’s contracture management: A meta-analysis. J. Orthop. Surg..

[33] Schulze S.M., Tursi J.P. (2014). Postapproval clinical experience in the treatment of Dupuytren’s contracture with collagenase clostridium histolyticum (CCH): The first 1000 days. Hand.

[34] Cao D., Li J., Lu Y., Huang Y., Chen B., Chen Z., Shen Y., Liu L., Wei Q. (2022). Efficacy and Safety of Collagenase Clostridium Histolyticum in the Treatment of Peyronie’s Disease: An Evidence-Based Analysis. Front. Med..

[35] Chen A.F., Lee Y.S., Seidl A.J., Abboud J.A. (2019). Arthrofibrosis and large joint scarring. Connect Tissue Res..

[36] Kim J., McFee M., Fang Q., Abdin O., Kim P.M. (2023). Computational and artificial intelligence-based methods for antibody development. Trends Pharmacol. Sci..

[37] Hamcerencu M., Desbrieres J., Popa M., Riess G. (2020). Thermo-sensitive gellan maleate/N-isopropylacrylamide hydrogels: Initial “in vitro” and “in vivo” evaluation as ocular inserts. Polym. Bull..

[38] Fertala J., Rivlin M., Wang M.L., Beredjiklian P.K., Steplewski A., Fertala A. (2020). Collagen-rich deposit formation in the sciatic nerve after injury and surgical repair: A study of collagen-producing cells in a rabbit model. Brain Behav..

[39] Schmitz H.C., Beer G.M. (2001). The toe-spreading reflex of the rabbit revisited—Functional evaluation of complete peroneal nerve lesions. Lab. Anim..

[40] Kang M.L., Im G.I. (2014). Drug delivery systems for intra-articular treatment of osteoarthritis. Expert Opin. Drug Deliv..

[41] Ogay V., Mun E.A., Kudaibergen G., Baidarbekov M., Kassymbek K., Zharkinbekov Z., Saparov A. (2020). Progress and Prospects of Polymer-Based Drug Delivery Systems for Bone Tissue Regeneration. Polymers.

[42] Rai M.F., Pham C.T. (2018). Intra-articular drug delivery systems for joint diseases. Curr. Opin. Pharmacol..

[43] Ilochonwu B.C., Urtti A., Hennink W.E., Vermonden T. (2020). Intravitreal hydrogels for sustained release of therapeutic proteins. J. Control. Release.

[44] Chen W., Li Z., Wang Z., Gao H., Ding J., He Z. (2020). Intraarticular Injection of Infliximab-Loaded Thermosensitive Hydrogel Alleviates Pain and Protects Cartilage in Rheumatoid Arthritis. J. Pain Res..

[45] Kadler K.E., Hojima Y., Prockop D.J. (1987). Assembly of collagen fibrils de novo by cleavage of the type I pC-collagen with procollagen C-proteinase. Assay of critical concentration demonstrates that collagen self-assembly is a classical example of an entropy-driven process. J. Biol. Chem..

